# Trend of suicide by self-immolation in a 13-year timeline: was the COVID-19 pandemic a potentially important stressor?

**DOI:** 10.3389/fpubh.2024.1234584

**Published:** 2024-02-21

**Authors:** Jelena V. Jeremic, Jovan M. Mihaljevic, Ivan L. J. Radosavljevic, Milana M. Jurisic, Branko J. Suđecki, Milan T. Stojicic, Milan D. Jovanović, Zorana Pavlovic, Kristina G. Radenovic, Nikola V. Milic, Vedrana Pavlovic, Natasa M. Milic, Marko S. Jovic

**Affiliations:** ^1^Faculty of Medicine, University of Belgrade, Belgrade, Serbia; ^2^Clinic for Burns, Plastic and Reconstructive Surgery, University Clinical Center of Serbia, Belgrade, Serbia; ^3^Clinic for Psychiatry, University Clinical Center of Serbia, Belgrade, Serbia; ^4^Department of Medical Statistics and Informatics, Faculty of Medicine, University of Belgrade, Belgrade, Serbia

**Keywords:** self-immolation, burns, mental health, suicide, COVID-19, joinpoint regression

## Abstract

**Introduction:**

Self-immolation is an uncommon way of attempting and committing a suicide, with a fatality rate of 80%. The risk factors in self-immolation victims vary depending on demographic characteristics, socio-economic and cultural factors as well as religious beliefs. Whether the COVID-19 pandemic was a potentially important stressor for self-immolation is still unknown, with insufficient studies examining this issue. Therefore, in this study, we aimed to examine the trend of self-immolation in a 13-year timeline, and the potential association of COVID-19 pandemic with the increase in the incidence and severity of self-immolation injuries in Serbia in 2021.

**Materials and methods:**

The study included hospitalized patients due to intentional burns caused by self-immolation in the period from January 1, 2008 to December 31, 2021. Joinpoint regression analysis was used for the analysis of continuous linear trends of self-immolation cases with change points.

**Results:**

While a rising trend was observed in the 2008–2013 time segment, followed by a decline in the upcoming 2013–2016 time segment, a significant increase reached its maximum during COVID-19 pandemic (2021), with annual percent change of 37.1% (*p* = 0.001). A significant increase in the median number of cases per year was observed during 2021 compared to the previous periods (7.5 vs. 2). Frequency of patients with a psychiatric diagnosis vs. those without a psychiatric diagnosis was significantly higher during than before the COVID-19 period (66.7 vs. 36.1%, *p* = 0.046).

**Conclusion:**

In our study, a significant increase in the frequency of suicide attempts by self-immolation during COVID-19 pandemic was noticed. There was also an increased frequency of pre-existing psychiatric illness among patients during the pandemic period. With limited high-quality data available, the study adds to a rising body of evidence for assessment of outcomes of the pandemic on mental health and recognition of stressors for self-immolation.

## 1 Introduction

More than 800,000 people die due to suicide every year, with self-harm being one of the leading causes of death worldwide (1). Most commonly described risk factors for suicidal behavior are previous suicide attempts, family history of suicidal behavior, mental illness, abuse trauma history (especially in childhood), anxiety disorder, as well as recent major stressor or impending crisis (2–7).

At the very beginning of the COVID-19 pandemic, many experts warned of the potential rise of suicide incidences with various reports emerging in the literature, though of debatable quality (8). Social isolation, fear of illness, death, job loss, sleep disorders, and lack of access to healthcare seem to have led to an increase in the frequency of psychiatric diagnoses and exacerbation of the previously existing mental disorders (9–11). Moreover, lifestyle changes resulting from COVID-19 have impacted symptom severity and recovery time, contributing to overall poor health outcomes (12). Recent studies have indicated that a lack of physical activity and a higher carbohydrate diet during isolation have led to poorer sleep patterns, indirectly affecting mental health (13). Finally, the association between the COVID-19 pandemic and frequency of suicidal thoughts, as well as the rise of suicide rates is being examined extensively, but only few have analyzed the incidence of self-immolation during the COVID-19 pandemic (14, 15).

Self-immolation is an uncommon way of attempting and committing a suicide. The demographic characteristics and risk factors in self-immolation victims vary depending on socio-economic factors, cultural factors as well as religious beliefs (14–18). Comprising from 0.4 to 40% of total burn center admissions worldwide, it is accompanied by high mortality of up to 80%, prolonged hospitalizations, frequent complications, as well as serious physical and psychological consequences in surviving patients (14, 15, 17, 18). Although not one of the common ways to attempt suicide in highly developed countries, it is very common in certain populations in the Middle East and Asia. In Europe, middle-aged men with a history of diagnosed psychiatric illness are most likely to choose this method of self-harm. In Asian countries, young, married women most often opt for self-immolation that is usually associated with cultural specificities, religion, and lifestyle (1, 2, 5, 7, 15–18).

In general, not many studies analyzed the incidence and factors related to self-immolation in the literature, with only two studies examining the impact of COVID-19 pandemic to the related topic. These studies conducted in Australia and Brazil found an increase in the incidence and severity of self-immolation injuries during COVID-19 (15, 18). While other historic events have been described to increase the risk of suicidal behavior, known risks of self-immolation related to recent events are limited and are yet to be explored. Therefore, in this study, we aimed to examine the trend of self-immolation in a 13-year timeline, and the potential association of COVID-19 pandemic with the increase in the incidence and severity of self-immolation injuries in Serbia in 2021.

## 2 Materials and methods

This retrospective cohort study was conducted at the Clinic for Burns, Plastic and Reconstructive Surgery of the University Clinical Center of Serbia (UCCS). The clinic serves as a national burn referral center, providing extensive burn injuries care for the entirety of Serbia’s population of 6,664,449 inhabitants. The studied population included patients hospitalized for burns caused by intentional self-immolation with suicidal intent between January 1, 2008, and December 31, 2021. The study inclusion criteria were inpatients treated for burn injuries with intentional self-immolation with suicidal intent as the confirmed cause of injury, whether self-reported or by a third-party (family member, eyewitness, or the accompanying medical care professional from the referring hospital). For cases where verbal confirmation was not possible, prior written or electronic patient data records were used with the clinical presentation and psychiatrist’s consultation to confirm the cause of injury. To ensure the accuracy of the supplementary medical data, all patient records were checked using ICD-10 coding system. ICD-10 codes checked for the inclusion into the study were: X70-X84 and/or Y87 (recent or past patient medical data), or F00-99, when combined with ICD-10 codes: T20-T27.7, T29.1-4. All individuals treated as outpatients, whether because of not meeting the Burns Unit admission criteria or per their own request, as well as the patients with insufficient background medical data combined with an unclear cause-of-injury scenario and/or non-distinct clinical presentation where self-immolation with suicide intent could not be confirmed were excluded from this study. After obtaining the Ethics Committee’s approval (number: 808/13, date 30 May 2022), the following patient data were extracted: general demographic data (age, sex), presence of psychiatric disorders, mechanism of injury, total body surface area (TBSA) affected (%), modified Baux score, presence of surgical intervention, length of hospital stay, and outcome. Regarding psychiatric disorders categorization, depression and bipolar disorders were classified as affective disorders, while psychotic disorders included schizophrenia and other related disorders. According to the mechanism of self-immolation, patients were categorized into two groups: (1) petrol, and (2) other flammable substances. Percentage of TBSA affected (%) was determined using the “Wallace rule of nines” which divides the body into regions that represent 9% or multiples of 9% of TBSA, and “the rule of palm” in which the surface area of the patient’s palm (including the fingers) is considered to be approximately 1% of the TBSA, as appropriate. Modified Baux scores were calculated as follows: age + TBSA (%) burned +17 (in case of inhalation injury). The outcome was depicted as the percentage of fatal outcomes in both cohorts.

### 2.1 Statistical analysis

Numerical data are presented as means with standard deviations, or medians with minimum and maximum values. Categorical variables are summarized by absolute numbers with percentages. Joinpoint regression analysis was used for the analysis of continuous linear trends of self-immolation cases with change points. The joinpoint regression analysis involves fitting a series of joined straight lines on a log scale to the trends in the annual self-immolation incidence (19). Differences in demographic, clinical and burn injury data before and during COVID-19 epidemic in Serbia were assessed by Student’s test or Mann–Whitney test for numerical data, according to data distribution, while Chi-square or Fisher’s exact test was used for categorical data. Fisher’s exact test was used in situations where the expected cell counts were fewer than 5. In all analyses, the significance level was set at 0.05. Statistical analysis was performed using Joinpoint Regression Program—Surveillance Research Program version 5.0.2 (20), and IBM SPSS statistical software (SPSS for Windows, release 25.0, SPSS, Chicago, IL, United States).

## 3 Results

**Figure 1:**
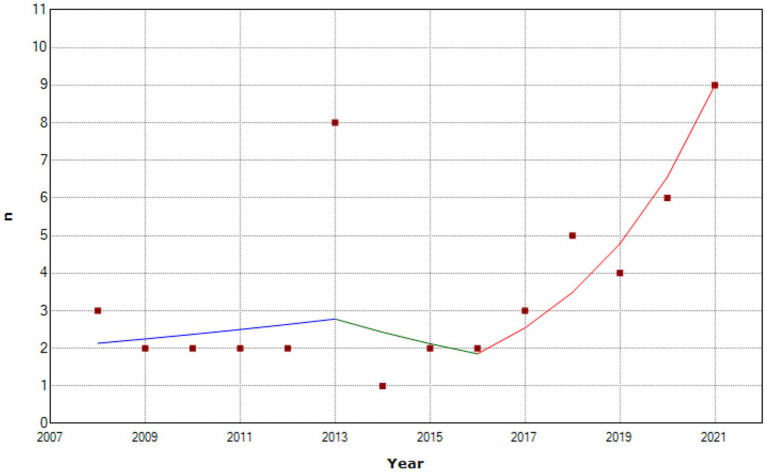
Number and trend of self-immolation suicide attempts per year from 2008 to 2021.

This study included 51 patients hospitalized due to burns caused by self-immolation in the period from 2008 to 2021 (Figure 1).

Joinpoint regression analysis of linear trends of self-immolation suicide attempts identified the following three segments: 2008 to 2013; 2013 to 2016; and 2016 to 2021, with two change points. While a rising trend was observed in the 2008–2013 time segment, followed by a decline in the upcoming 2013–2016 time segment, a significant increase reached its maximum during COVID-19 pandemic (2021), with annual percent change of 37.1% (*p* = 0.001) (Table 1). A significant increase in the median number of cases per year was observed during 2021 compared to the previous periods (7.5 vs. 2).

**Table 1:** Join point regression analysis of self-immolation suicide attempts 2008–2021.

Segment	Lower endpoint	Upper endpoint	APC	Lower CI	Upper CI	Test statistic (*t*)	Prob > |*t*|
1	2008	2013	5.4	−8.1	20.9	0.9	0.383
2	2013	2016	−12.6	−52.7	61.4	−0.5	0.610
3	2016	2021	37.1^*^	19.5	57.3	5.6	0.001

Based on the time of proclamation of COVID-19 pandemic, patients were divided into two groups: pre-COVID-19 (*n* = 36) and COVID-19 (*n* = 15). In the COVID-19 period, an increase in the number of cases was recorded in contrast to the pre-COVID period (median number of cases per year 7.5 vs. 2). Demographic, clinical, and burn injury data for both groups are presented in Table 2.

**Table 2:** Demographic, clinical and burn injury data.

	Pre-COVID	COVID	*p*
	*n* = 36	*n* = 15	
Age, mean ± SD	41.6±14.8	49.7±14.2	0.078
Gender, *n* (%)			0.783
Male	18 (50.0)	6 (40.0)	
Female	18 (50.0)	9 (60.0)	
Psychiatric history, *n* (%)	13 (36.1)	10 (66.7)	0.046
Affective disorder, *n* (%)	4 (11.1)	5 (33.3)	0.058
Mechanism of injury, *n* (%)			0.284
Petrol	27 (75.0)	9 (60.0)	
Other	9 (25.0)	6 (40.0)	
TBSA, median (range)	30 (1–95)	30 (5–98)	0.893
Baux score, median (range)	86 (36–135)	83 (31–125)	0.700
Surgery, *n* (%)	24 (66.7)	8 (53.3)	0.370
Mortality, *n* (%)	16 (44.4)	9 (60.0)	0.311
Length of hospital stay, median (range)	21 (1–204)	10 (1–121)	0.116

**Figure 2:**
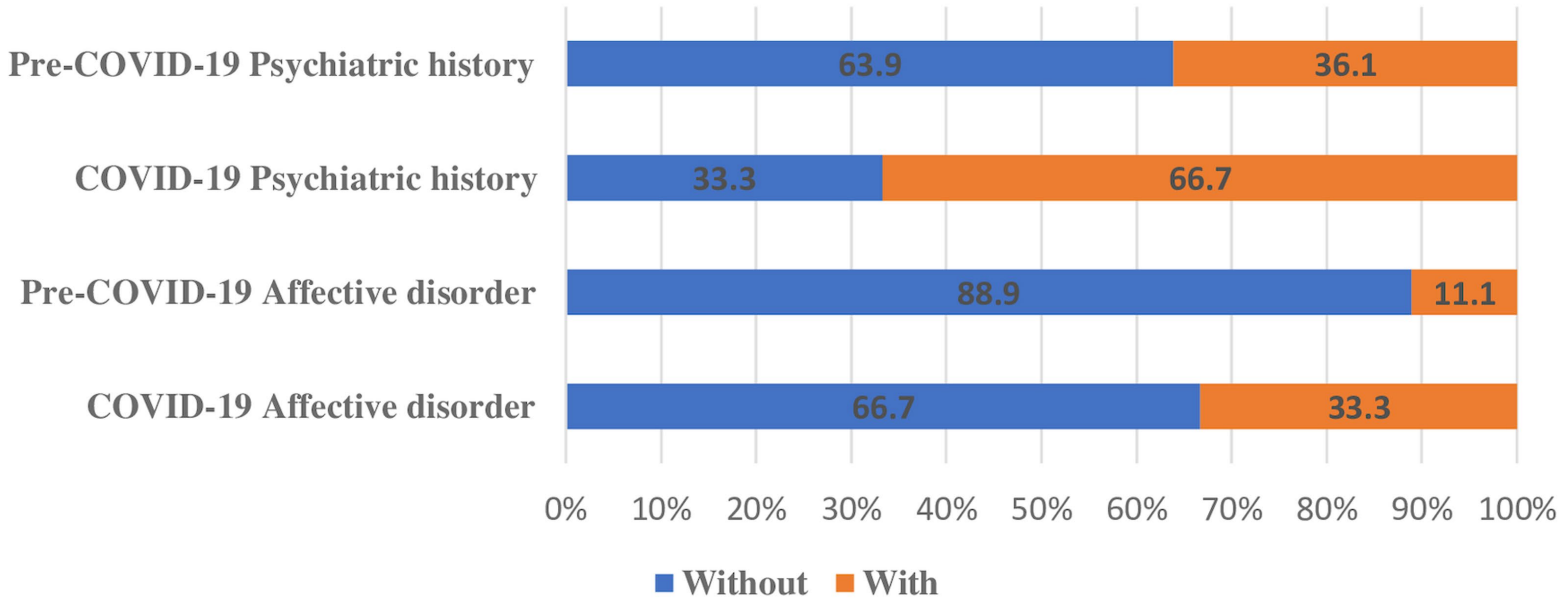
Self-immolation patients according to psychiatric history and presence of affective disorder before and during Covid-19 pandemic.

Patients hospitalized in the COVID-19 period were older than patients hospitalized in pre-COVID period (49.7 vs. 41.6 years). History of psychiatric illness, such as an established diagnosis prior to the event, was present in 45.1% of patients. Frequency of patients with a psychiatric diagnosis vs. those without a psychiatric diagnosis was significantly higher during than before the COVID-19 period (66.7 vs. 36.1%, *p* = 0.046). In particular, there was an increase in affective psychiatric diagnoses during the COVID-19 period (33.3 vs. 11.1%) (Figure 2).

**Figure 3:**
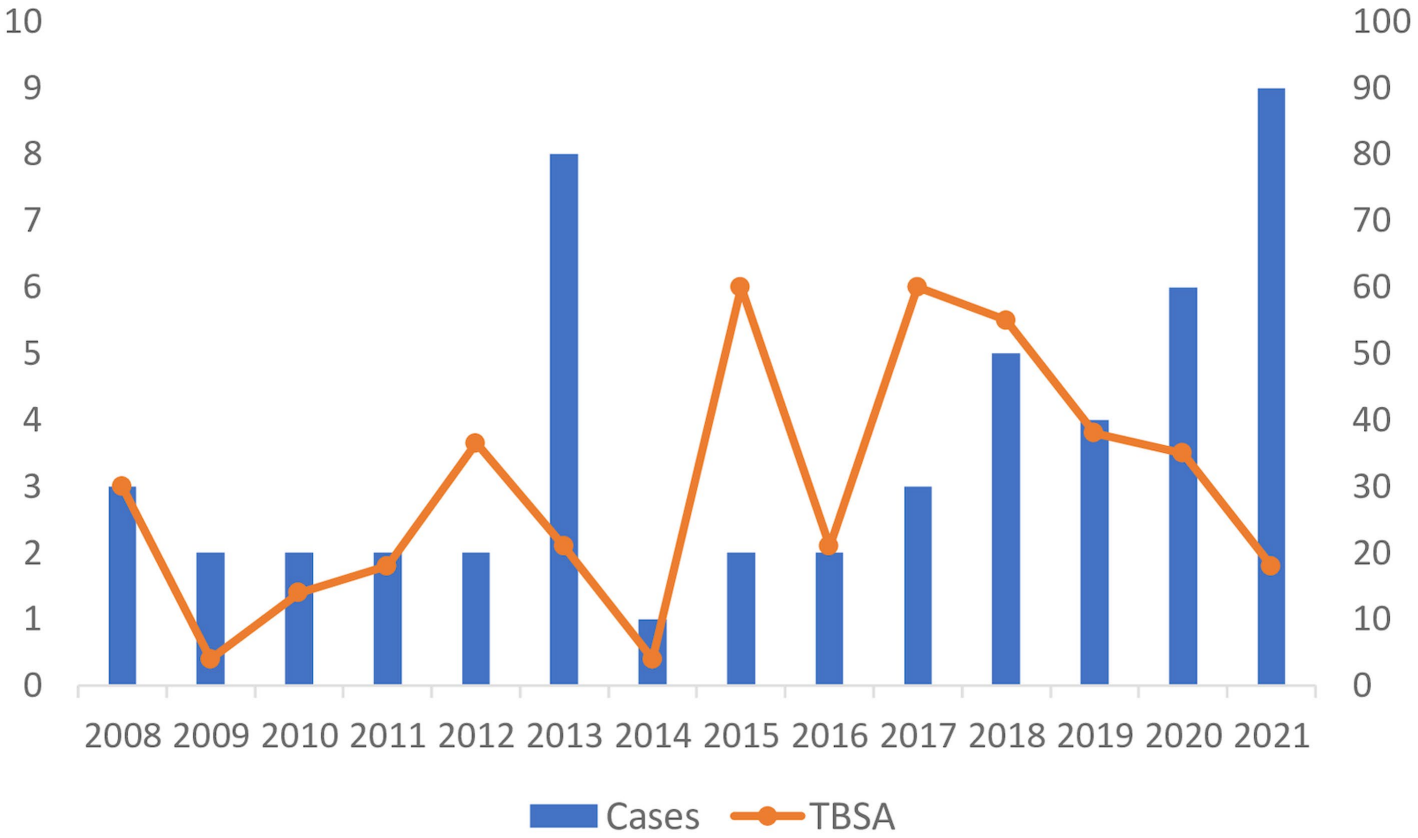
Total body surface area of self-immolation patients from 2008 to 2021.

**Figure 4:**
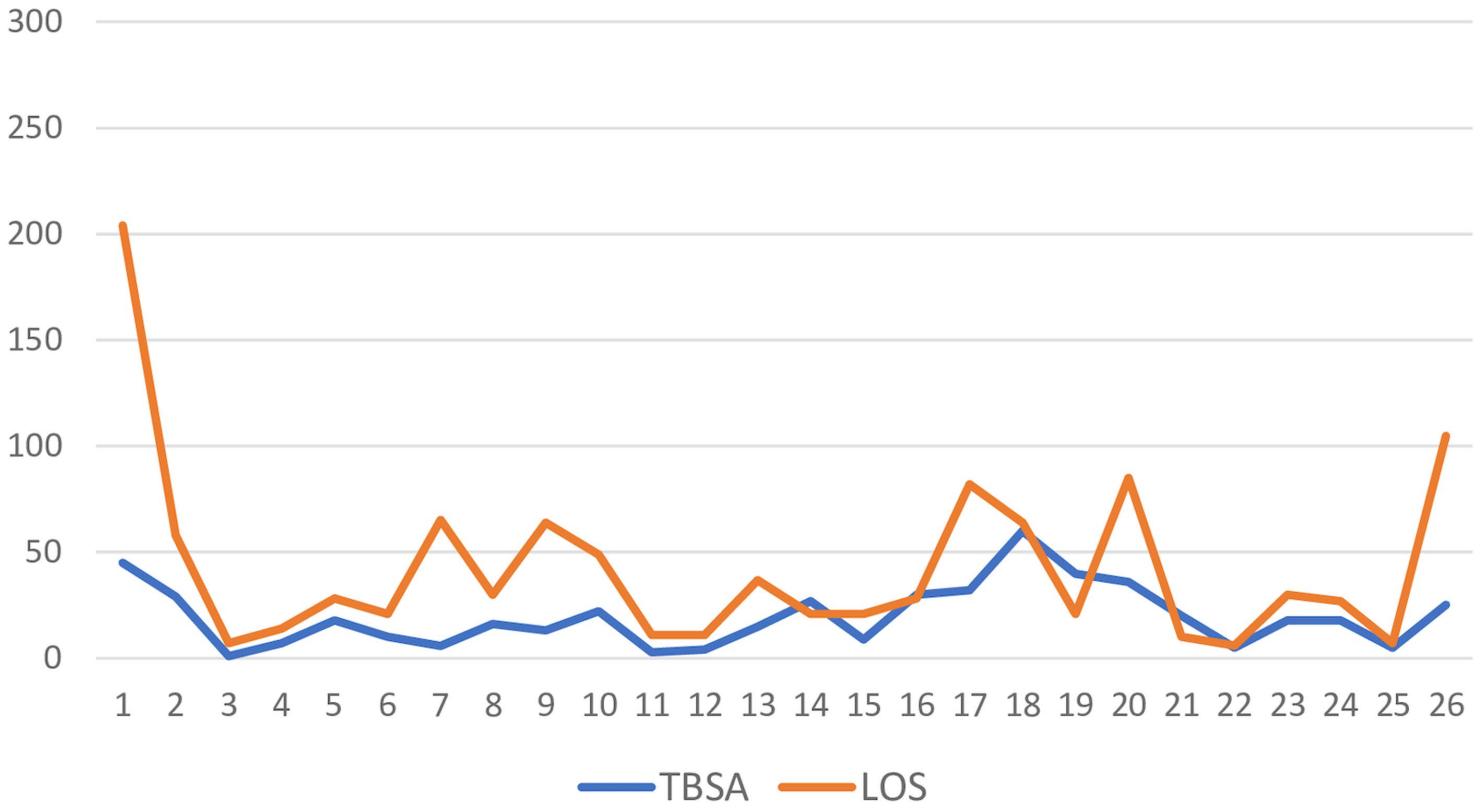
Total body surface area according to length of stay in survived self-immolation patients.

The most common mechanism of self-immolation in both groups was gasoline pouring (COVID-19 60% vs. pre-COVID-19 75%), with an increase in frequency of other mechanisms of self-immolation during COVID-19 (25.0 vs. 40.0%). Although the total body surface area (TBSA) affected and modified Baux score did not change significantly during versus before COVID-19, a decrease in number of surgeries and shorter length of hospitalization was observed during the COVID-19 period (53.3 vs. 66.7%, 10 vs. 21 days, respectively) (Figure 3). Total body surface area according to length of stay in survived self-immolation patients is presented in Figure 4. The number of patients with lethal outcome increased during the COVID-19 period (60.0 vs. 44.4%) (Table 2).

## 4 Discussion

During the observed period from 2008 to 2021, two change points of increased self-immolation frequency were revealed: the period following the World economic crisis (2013) and during the COVID-19 pandemic (2021).

The impact of COVID-19 on suicide rates has been excessively studied, published as well as criticized (8). A review by Pathirathna et al. (21) reported an increasing trend of suicidal attempts during the COVID-19 pandemic compared to the rates reported in previous years. Additionally, studies that have addressed the frequency of suicidal thoughts also report an increase during the pandemic (22, 23). In contrast, Appleby and colleagues report stable suicide rates in England in the first 7 months after the first national lockdown, the results comparable to other high-income countries (24). In comparison to other historic major outbreaks, a group of authors found an increase in death by suicide during the Spanish Flu pandemic, occurring in 1918, with additional evidence supported by Yip et al. regarding a significant increase in suicide deaths among people older than 65 during the SARS outbreak in Hong Kong in 2003 (25, 26). Still, systematic reviews of suicide rates during major international outbreaks (SARS, Influenza, Ebola, including COVID-19) describes little evidence for an increased risk of suicide during the analyzed viral outbreaks (10, 27). However, there is an agreement in scientific community that there is a lack of evidence to establish this association unequivocally (27, 28).

Only several studies explored the relationship between the COVID-19 pandemic and self-immolation (27, 28). Our study found a significant increase in the median number of cases per year during 2021 compared to the previous periods (7.5 vs. 2). Similar results were reported by Marques et al. (15), with an increase in self-inflicted burn injuries in the pandemic period, from December 2019 to June 2020, at the Burn Unit from University of São Paulo, Brazil. A study by Jackson and colleagues found an increase in 2020 in both the frequency and severity of self-inflicted burn injuries in New South Wales. The authors reported 18 cases of self-immolation in 2020, compared to an average of 10 cases per year. Similar to our study, an increased presence of psychiatric disorders was found as a major contributing factor (14). In our study frequency of patients with a psychiatric diagnosis compared to those without a psychiatric diagnosis was higher during than before the COVID-19 pandemic (66.7 vs. 41.7%, respectively). Affective psychiatric disorders, mainly depression and bipolar disorder were mostly prevalent among all psychiatric diagnoses. Psychological disorders such as personality disorders, schizophrenia, in addition to economic and social factors were found to be important factors related to self-immolation in previous studies, while one study on subjects who attempted self-immolation concluded that schizophrenia, depression, and personality disorder were diagnosed in 71% of participants (29, 30). Additionally, authors from a Burn center in Spain found that 60.3% of patients admitted due to self-immolation had history of psychosis, depression or schizophrenia (31). While COVID-19 pandemic was proven as a significant cause of psychological distress in the general population, consequences of isolation, anxiety, fear of illness and death, lack of sustenance, lack of access to health care and many other factors that accompanied the COVID-19 pandemic played a rather unfavorable role in exacerbating the existing mental illnesses, subsequently leading to the increase of suicide attempts among psychiatric patients by self-immolation (9–11, 22, 23, 32–34). Furthermore, these results are consistent with recent findings that reported increased frequency of pre-existing psychiatric illness among admitted patients during the pandemic period due to self-immolation (14, 15). Most importantly, survivors of self-inflicted burn injuries face an increased risk of recurrent suicide attempts, due to the side effects of burns such as disfigurement and disability (35).

The detrimental effects of economic downturn following the pandemic on mental health and suicide have been increasingly recognized and studied in the literature. Numerous studies and review papers have linked suicide behavior to financial stressors, including unemployment and financial insecurity (36–39). A recent European study emphasized the role of debt and job loss in suicide rates (40). Further investigations, such as an analysis of 675 urban suicides in the United States from 1997 to 2000, revealed economic strain in 9%, with key stressors being job loss and home loss (41). In a Welsh study from 2002 to 2005, debt and employment issues contributed to 23% of male suicides (42). Furthermore, suicide rates related to one of the most pernicious economic crises, the Great Depression in the 1930s, have been reported by Tapia et al., showing an increase in suicide rates in the United States, while Varnik et al. reported evidence of its impact on the European countries with increase of suicide rates in Estonia in the early 1930s (43, 44).

In comparison to other countries with more stable economies, the recession following 2007 and 2008 had a rather heavier impact in Serbia with the lowest growth of the Serbian Gross Domestic Product (GDP) when compared to other western Balkan countries at that time (45, 46). The World economic crisis led to an increase of unemployment, fear of job loss, and lower quality of life among Serbian citizens. It could be assumed that economic instability and existential anxiety have had an impact on mental health, especially in vulnerable groups, which could subsequently lead to an increased rate of suicide attempts. However, data regarding self-immolation in relationship to economical factors in the literature is limited. A rise in the incidences of self-immolation in the aftermath of the economic crisis has been observed in our study, though without statistical significance. These findings could be limited by a small population sample but are worth reflecting upon in association to the similar detrimental socio-economic effects of the COVID-19 pandemic as a potential stressor. Further research is necessary on a larger population in a multicentric design for better comprehension of these possibly causal relationships.

### 4.1 Limitations

This study is limited by its retrospective nature and the potential biases associated with its study design. Additional limitations include insufficiently detailed data on the immediate cause, i.e., the stressor leading to a suicide attempt, detailed data on the history of previous psychiatric treatments, as well as patients’ socio-economic conditions. The studied population is susceptible to a selection bias because of inclusion of patients referred to and hospitalized in a tertiary institution. Furthermore, exclusion of self-immolation suicide attempters who died on-site or before referral to our Clinic may influence the self-immolation suicide rates and corresponding mortality.

## 5 Conclusion

In our study, a significant increase in the frequency of suicide attempts by self-immolation during COVID-19 pandemic was noticed. There was also an increased frequency of pre-existing psychiatric illness among subjects during the pandemic period. With limited high-quality data available, the study adds to a rising body of evidence for assessment of outcomes of the pandemic on mental health and recognition of stressors for self-immolation.

## Data availability statement

The original contributions presented in the study are included in the article/supplementary material, further inquiries can be directed to the corresponding author.

## Ethics statement

The studies involving humans were approved by the Institutional Review Board of University Clinical Center of Serbia (number: 808/13, date 30 May 2022). The studies were conducted in accordance with the local legislation and institutional requirements. The ethics committee/institutional review board waived the requirement of written informed consent for participation from the participants or the participants’ legal guardians/next of kin because this was a retrospective study based on patient medical records with adequate patient anonymization.

## Author contributions

JJ, JM, IR, MJu, BS, MS, MJova, ZP, KR, NiM, NaM, VP, and MJovi contributed to the study conception and design. JJ and JM are in charge of the main idea and are the guarantors of integrity of the entire study. JJ, JM, MJovi, MS, MJova, MJu, BS, IR, KR, and ZP are in charge of the study concepts, design, manuscript preparation, and editing. MJovi is in charge of supervision and project administration. Material preparation and editing were performed by JM, MJu, BS, KR, and IR. Statistical analysis, data processing, and graphical data presentation were performed by NaM, NiM, and VP. All authors contributed to the article and approved the submitted version.
